# Signal mining and analysis of ripretinib adverse events: a real-world pharmacovigilance analysis based on the FAERS database

**DOI:** 10.3389/fphar.2025.1481114

**Published:** 2025-02-26

**Authors:** Ye Hu, Linlin Zhang, Qineng Gong, Lei Huang, Cunlin Yin, Yang Miao, Hui Wu

**Affiliations:** ^1^ Department of Pharmacology, The First People’s Hospital of Yancheng, Yancheng, Jiangsu, China; ^2^ Medical Research Center, Affiliated Hospital 2 of Nantong University, Nantong, Jiangsu, China

**Keywords:** ripretinib, adverse event, FAERS, disproportionality analysis, pharmacovigilance

## Abstract

**Background:**

Ripretinib is a tyrosine kinase inhibitor indicated for the treatment of adult patients with advanced gastrointestinal stromal tumors (GISTs) who have previously received treatment with at least three kinase inhibitors. The objective of this study was to evaluate adverse events(AEs) associated with ripretinib using data from the FDA Adverse Event Reporting System (FAERS) database.

**Methods:**

Individual case safety reports (ICSRs) related to of ripretinib from 2020 Q2 to 2024 Q2 were extracted from the FAERS database. This study used the reporting odds ratio (ROR), proportional reporting ratio (PRR), Bayesian confidence propagation neural network (BCPNN), and multi-item gamma Poisson shrinker (MGPS) for disproportionality analysis. In addition, this research also performed a descriptive analysis of the time-to-onset (TTO) of AEs related to ripretinib.

**Results:**

A total of 3,513 ICSRs with ripretinib as the primary suspect (PS) were retrieved from the FAERS database. At the preferred term(PT) level, this study detected 116 positive AEs. Common AEs included alopecia, constipation, muscle spasms, dry skin, decreased appetite. Notably, unexpected AEs such as pleural mass, blood magnesium abnormal, blood potassium abnormal, hepatic lesion, and liver abscess were also observed. The median time to onset of ripretinib-related AEs was 102 days (29–254 days), with the majority of AEs occurring during the first month of treatment.

**Conclusion:**

This study identified some known AEs associated with ripretinib and discovered unexpected AEs, providing preliminary insights into its safety in the real world. This information is valuable for clinical monitoring and the safe use of ripretinib.

## 1 Introduction

Ripretinib is a novel small-molecule switch-controlled tyrosine kinase inhibitor that demonstrates potent inhibition of a broad spectrum of kinases, including KIT (stem cell factor receptor) and PDGFRA(platelet-derived growth factor receptor alpha) (Smith et al., 2019; Gupta et al., 2021). Additionally, ripretinib inhibits several other kinases *in vitro*, including B-Raf proto-oncogene, serine/threonine kinase (BRAF), vascular endothelial growth factor receptor-2 (VEGFR2), platelet-derived growth factor receptor beta (PDGFRB), and TEK receptor tyrosine kinase (TIE2) (Smith et al., 2019). It has been approved by the U.S. Food and Drug Administration (FDA) and the European Medicines Agency (EMA) for the treatment of adult patients with advanced gastrointestinal stromal tumors (GISTs) who have previously undergone treatment with three or more kinase inhibitors (Fung and Shirley, 2022). Clinical studies have demonstrated that ripretinib significantly delays disease progression and improves overall survival in these patients (Blay et al., 2020; Von Mehren et al., 2021).

However, despite the therapeutic efficacy of ripretinib, its widespread use inevitably leads to adverse events (AEs) in patients. Common AEs reported in at least 20% of participants in Phase III clinical trials involving ripretinib include alopecia, myalgia, nausea, fatigue, palmar-plantar erythrodysesthesia syndrome, and diarrhea (Blay et al., 2020). In addition, the drug label for ripretinib lists other adverse events, such as hypertension, new primary cutaneous cancers, and photosensitivity. Given these risks, there is an urgent need for data mining to assess ripretinib’s post-marketing safety profile.

The FDA Adverse Event Reporting System (FAERS) is one of the largest global repositories of pharmacovigilance data, encompassing AE reports from diverse sources, including physicians, pharmacists, consumers, and lawyers (Mikami et al., 2021; Setyawan et al., 2021). It serves as a critical tool for the FDA in conducting comprehensive post-marketing drug safety evaluations. The purpose of this study is to evaluate the real-world safety of ripretinib through the FAERS database and provide recommendations for its safe clinical use.

## 2 Materials and methods

### 2.1 Data sources and management

Ripretinib has been licensed for marketing by the FDA since May 2020. In this study, we utilized the FAERS database to perform a retrospective safety analysis of individuals using ripretinib between Q2 2020 and Q2 2024. The FAERS database report contains seven datasets: demographic and administrative information (DEMO), drug information (DRUG), adverse drug reaction information (REAC), patient outcome information (OUTC), reporting source information (RPSR), date of treatment initiation and end date of reported medication (THER), and medication administration indications (INDI). We organized the reports by Case Identifiers (CASEIDs), FDA Receipt Date (FDA_DT), and Primary Identifiers (PRIMARYID). For reports with the same CASEID, we retained the report with the latest FDA_DT value, and for reports with both the same CASEID and FDA_DT, we retained the report with the largest PRIMARYID value. This approach allowed for the identification and elimination of duplicate reports.

**FIGURE 1:**
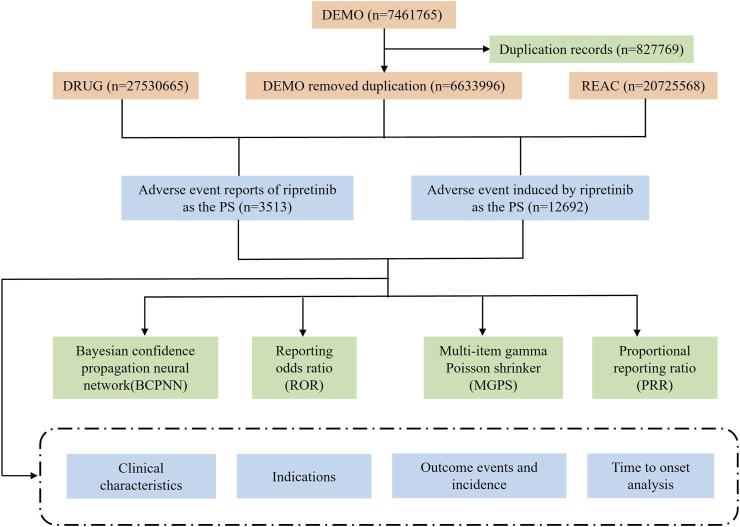
Flowchart of the entire study.

The FAERS database was searched using the brand name “Qinlock” and the generic name “ripretinib”, and only reports where the target drug was the primary suspect (PS) were extracted. The reported AEs associated with ripretinib were classified at the system organ class (SOC) and preferred term (PT) levels using the Medical Dictionary for Regulatory Activities (MedDRA, version 27.0). Figure 1 illustrates the entire analytical procedure.

### 2.2 Time to onset (TTO) analysis

The TTO of ripretinib-associated AEs was calculated by subtracting the date of drug initiation from the date of AE onset. The median and interquartile range (IQR) were used to describe the duration of the episodes, excluding reports with inaccurate or missing date entries, as well as AEs that occurred prior to the initiation of therapy.

### 2.3 Statistical analysis

Disproportionality analysis is a widely used method in pharmacovigilance. We used the reporting odds ratio (ROR), proportional reporting ratio (PRR), bayesian confidence propagation neural network (BCPNN), and multi-item gamma Poisson shrinker (MGPS) for signal identification. ROR and PRR are classified as frequency-based approaches, indicating high sensitivity but low specificity. BCPNN and MGPS are Bayesian algorithms that can handle complex variables but provide low sensitivity in signal detection (Zou et al., 2023). We used the four algorithms to guarantee that the study’s results were stable and reliable. In this study, an adverse event term was considered significant only if it met the threshold criteria of all four algorithms. Supplementary Table S1 shows the formulas and criteria for each of the four algorithms.

To evaluate the impact of gender and age on ripretinib-induced AEs, we used the ROR method, with the exact formula provided in Supplementary Table S2. The *p*-value was calculated using the chi-square (χ^2^) test based on a 2 × 2 contingency table. When ROR >1 and *p* < 0.05, it indicated that females or patients over 60 were more likely to report a specific AE. Conversely, when ROR <1 and p < 0.05, males or patients under 60 were more likely to report the AE.

## 3 Results

### 3.1 Descriptive characteristics

During the study period, a total of 7,461,765 ICSRs were submitted to the FAERS database, of which 3,513 were ICSRs with ripretinib as the PS. The clinical characteristics of ripretinib-associated AEs are shown in Table 1. Among all ICSRs, there were more males (54.00%) than females (44.04%). In terms of age, patients between 65 and 85 accounted for the majority of ICSRs (24.68%).

**TABLE 1:** Clinical characteristics of ripretinib-related reports in the FAERS database.

Characteristics	Case Number, n	Proportion, %
Overall	3,513	
Gender		
Female	1,547	44.04
Male	1897	54.00
Missing	69	1.96
Age		
<18	2	0.06
18∼64	599	17.05
65∼85	867	24.68
>85	53	1.51
Missing	1992	56.70
Outcome		
Hospitalization-Initial or Prolonged	603	17.16
Death	349	9.93
Life-Threatening	12	0.34
Other Serious (Important Medical Event)	390	11.10
Missing	2,159	61.46
Indications (top five)		
Gastrointestinal stromal tumour	2,548	72.53
Product used for unknown indication	742	21.12
Neoplasm malignant	75	2.13
Gastric cancer	26	0.74
Malignant connective tissue neoplasm	25	0.71
Report recorder		
Consumer	2144	61.03
Health Professional	706	20.10
Physician	578	16.45
Pharmacist	80	2.28
Missing	5	0.14
Reported countries (top five)		
United States	3,276	93.25
France	57	1.62
Canada	47	1.34
China	25	0.71
Germany	15	0.43

**FIGURE 2:**
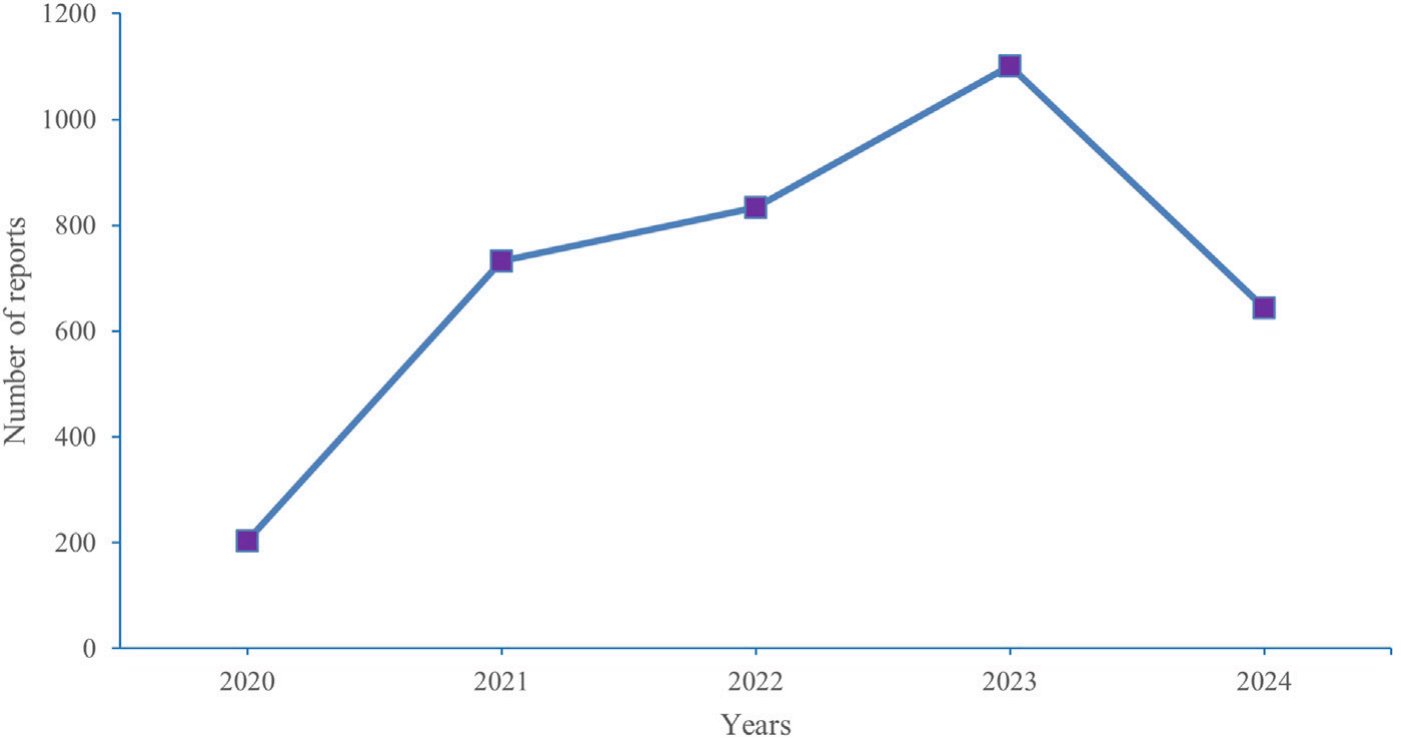
Annual distribution of adverse events related to ripretinib Reported from 2020 (Q2) to 2024 (Q2).

The most common serious outcomes were hospitalization (initial or prolonged) (17.16%) and death (9.93%), with death likely being more closely associated with cancer progression. Regarding reported indications, GIST (72.53%) was the primary indication. The United States accounted for the highest number of reports (93.25%), with the majority of reporters being consumers (61.03%) and healthcare professionals (20.10%). In terms of reporting years, there were 203 reports in 2020, 732 reports in 2021, 833 reports in 2022, 1,101 reports in 2023, and 644 reports in 2024. Figure 2 depicts the historical trend in the number of reports.

### 3.2 SOC signals

**FIGURE 3:**
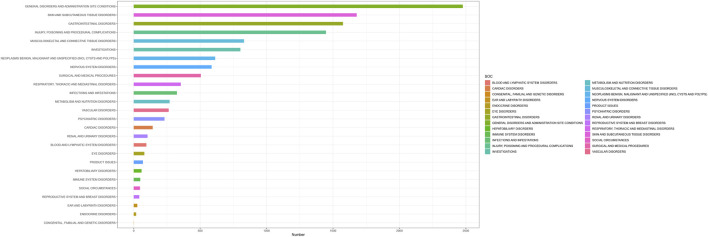
Distribution of the number of the system organ classifications (SOC).

By analyzing the AE reports of ripretinib, it was found that a total of 26 SOCs were involved in drug-related AEs. Figure 3 shows the number of cases occurring in different SOC, sorted by the number of AEs in the SOCs. Table 2 lists the signal intensity of ripretinib in different SOCs. According to the study’s findings, the top ranked SOCs were general disorders and administration site conditions (n = 2,477), skin and subcutaneous tissue disorders (n = 1,678), gastrointestinal disorders (n = 1,575), injury, poisoning, and procedural complications (n = 1,447), and musculoskeletal and connective tissue disorders (n = 830). The top five SOCs, ranked by ROR signal intensity, were surgical and medical procedures (ROR = 2.79), skin and subcutaneous tissue disorders (ROR = 2.75), gastrointestinal disorders (ROR = 1.66), musculoskeletal and connective tissue disorders (ROR = 1.26), and neoplasms benign, malignant, and unspecified (incl cysts and polyps) (ROR = 1.22).

**TABLE 2:** Signal intensity of ripretinib reports at the system organ classification (SOC) level in the FAERS database.

System organ class (SOC)	N	ROR (95% two-sided CI)	PRR (χ^2^)	EBGM (EBGM05)	IC (IC025)
Surgical and medical procedures	505	2.79 (2.55–3.05)*	2.72 (555.9)*	2.72 (2.52)*	1.44 (-0.22)
Skin and subcutaneous tissue disorders	1,678	2.75 (2.62–2.90)*	2.52 (1,623.91)*	2.52 (2.41)*	1.33 (-0.33)
Gastrointestinal disorders	1,575	1.66 (1.58–1.75)*	1.58 (364.74)	1.58 (1.51)	0.66 (-1.01)
Musculoskeletal and connective tissue disorders	830	1.26 (1.18–1.36)*	1.25 (43.02)	1.25 (1.18)	0.32 (-1.35)
Neoplasms benign, malignant and unspecified (incl cysts and polyps)	613	1.22 (1.13–1.32)*	1.21 (23.27)	1.21 (1.13)	0.27 (-1.39)
General disorders and administration site conditions	2,477	1.13 (1.08–1.18)*	1.11 (30.28)	1.11 (1.07)	0.14 (-1.52)
Vascular disorders	263	1.13 (1.00–1.28)	1.13 (3.86)	1.13 (1.02)	0.17 (-1.49)
Metabolism and nutrition disorders	270	1.13 (1.00–1.27)	1.13 (3.85)	1.13 (1.02)	0.17 (-1.50)
Investigations	803	1.07 (0.99–1.15)	1.06 (3.24)	1.06 (1.00)	0.09 (-1.58)
Injury, poisoning and procedural complications	1,447	0.90 (0.85–0.95)	0.91 (14.39)	0.91 (0.87)	−0.13 (-1.80)
Social circumstances	47	0.76 (0.57–1.01)	0.76 (3.50)	0.76 (0.60)	−0.39 (-2.06)
Nervous system disorders	586	0.63 (0.58–0.68)	0.64 (125.23)	0.64 (0.60)	−0.64 (-2.30)
Respiratory, thoracic and mediastinal disorders	354	0.61 (0.54–0.67)	0.62 (88.56)	0.62 (0.56)	−0.7 (-2.36)
Cardiac disorders	143	0.59 (0.50–0.70)	0.59 (40.20)	0.6 (0.52)	−0.75 (-2.42)
Hepatobiliary disorders	59	0.57 (0.44–0.74)	0.57 (19.01)	0.57 (0.46)	−0.8 (-2.47)
Reproductive system and breast disorders	42	0.57 (0.42–0.78)	0.57 (13.29)	0.57 (0.45)	−0.8 (-2.46)
Endocrine disorders	18	0.53 (0.33–0.84)	0.53 (7.64)	0.53 (0.36)	−0.92 (-2.59)
Ear and labyrinth disorders	27	0.52 (0.35–0.75)	0.52 (12.24)	0.52 (0.38)	−0.95 (-2.62)
Renal and urinary disorders	104	0.45 (0.37–0.55)	0.46 (67.84)	0.46 (0.39)	−1.13 (-2.79)
Blood and lymphatic system disorders	95	0.44 (0.36–0.53)	0.44 (68.58)	0.44 (0.37)	−1.18 (-2.85)
Infections and infestations	325	0.43 (0.38–0.48)	0.44 (243.22)	0.44 (0.40)	−1.18 (-2.84)
Immune system disorders	49	0.34 (0.26–0.45)	0.34 (63.22)	0.34 (0.27)	−1.55 (-3.22)
Psychiatric disorders	232	0.33 (0.29–0.37)	0.34 (315.97)	0.34 (0.30)	−1.56 (-3.23)
Eye disorders	80	0.33 (0.26–0.41)	0.33 (111.13)	0.33 (0.27)	−1.6 (-3.27)
Product issues	69	0.29 (0.23–0.36)	0.29 (122.61)	0.29 (0.24)	−1.79 (-3.45)
Congenital, familial and genetic disorders	1	0.03 (0.00–0.21)	0.03 (31.22)	0.03 (0.01)	−5.05 (-6.71)

ROR, reporting odds ratio; CI, confidence interval; PRR, proportional reporting ratio; χ^2^, chi-squared; EBGM, empirical Bayesian geometric mean; EBGM, 05, the lower limit of 95% CI, of EBGM; IC, information component; IC025, the lower limit of 95% CI, of the IC. N: Case numbers. *Indicates statistically significant signals in algorithm.

### 3.3 PT signals

Four algorithms were used to detect drug-induced AE signals, and 116 AE signals that met the threshold requirements of all four algorithms simultaneously, as shown in Supplementary Table S3.

The top 50 AEs were ranked at the PT level based on AE frequency and signal intensity determined by the ROR method, shown in Tables 3, 4. Alopecia (n = 436), constipation (n = 204), muscle spasms (n = 188), dry skin (n = 164), decreased appetite (n = 154), and hypertension (n = 148) were the common AEs in our study, after excluding medication doses and disease progression. These findings are consistent with the details provided in the drug labels and clinical trials. AEs with significant risk signals included hepatic embolisation (ROR = 288.06, PRR = 287.99, EBGM = 244.94, IC = 7.94), pleural mass (ROR = 204.06, PRR = 204.00, EBGM = 181.44, IC = 7.50), tumour compression (ROR = 173.75, PRR = 173.61, EBGM = 157.02, IC = 7.29), hyperkeratosis (ROR = 116.02, PRR = 115.01, EBGM = 107.51, IC = 6.75), drain site complication (ROR = 99.94, PRR = 99.92, EBGM = 94.21, IC = 6.56). In addition, data mining revealed some important AEs not mentioned in ripretinib’s product labels, such as pleural mass, blood magnesium abnormal, granulocyte count increased, blood urea abnormal, blood chloride decreased, prostatomegaly, hepatic lesion, small intestinal perforation, liver abscess, dry age-related macular degeneration, blood potassium decreased, blood glucose abnormal, hypersomnia. This analysis uncovered additional AEs, highlighting the need for increased safety monitoring of ripretinib.

**TABLE 3:** The top 50 AEs of ripretinib ranked by the frequency at PTs levels.

PTs	N	ROR (95% two-sided CI)	PRR (χ^2^)	EBGM (EBGM05)	IC (IC025)
SOC: Gastrointestinal disorders					
Constipation	204	4.66 (4.06–5.36)	4.61 (576.16)	4.60 (4.09)	2.20 (0.53)
Gingival bleeding	33	15.59 (11.06–21.97)	15.55 (445.15)	15.41 (11.57)	3.95 (2.28)
SOC: General disorders and administration site conditions					
Disease progression	246	9.69 (8.53–10.99)	9.52 (1867.97)	9.47 (8.52)	3.24 (1.58)
Adverse event	203	14.56 (12.67–16.74)	14.34 (2,500.71)	14.23 (12.66)	3.83 (2.16)
SOC: Injury, poisoning and procedural complications					
Extra dose administered	329	38.16 (34.16–42.63)	37.20 (11,338.80)	36.39 (33.17)	5.19 (3.52)
Underdose	270	21.10 (18.69–23.83)	20.68 (4,997.58)	20.43 (18.46)	4.35 (2.69)
Product dose omission in error	44	4.39 (3.26–5.90)	4.37 (114.30)	4.36 (3.41)	2.13 (0.46)
Product administration interrupted	13	3.34 (1.94–5.76)	3.34 (21.29)	3.34 (2.12)	1.74 (0.07)
SOC: Investigations					
Blood iron decreased	31	10.23 (7.18–14.56)	10.20 (255.82)	10.15 (7.55)	3.34 (1.68)
Blood bilirubin increased	30	7.76 (5.42–11.11)	7.75 (175.46)	7.71 (5.71)	2.95 (1.28)
Red blood cell count decreased	21	3.27 (2.13–5.02)	3.26 (32.93)	3.26 (2.28)	1.70 (0.04)
Blood potassium decreased^a^	19	3.51 (2.24–5.51)	3.51 (34.05)	3.51 (2.4)	1.81 (0.14)
Blood pressure abnormal	16	3.42 (2.09–5.59)	3.42 (27.31)	3.41 (2.26)	1.77 (0.10)
Blood sodium decreased	12	3.72 (2.11–6.57)	3.72 (23.84)	3.72 (2.31)	1.89 (0.23)
Blood glucose abnormal^a^	12	3.45 (1.96–6.08)	3.45 (20.84)	3.44 (2.14)	1.78 (0.12)
Blood alkaline phosphatase increased	11	3.59 (1.99–6.49)	3.59 (20.50)	3.58 (2.18)	1.84 (0.17)
SOC: Metabolism and nutrition disorders					
Decreased appetite	154	3.27 (2.79–3.83)	3.24 (238.87)	3.24 (2.83)	1.69 (0.03)
SOC: Musculoskeletal and connective tissue disorders					
Muscle spasms	188	6.13 (5.31–7.08)	6.06 (792.65)	6.04 (5.35)	2.59 (0.93)
Myalgia	133	4.88 (4.11–5.79)	4.83 (404.24)	4.82 (4.18)	2.27 (0.60)
SOC: Neoplasms benign, malignant and unspecified (incl cysts and polyps)					
Neoplasm progression	223	20.98 (18.36–23.97)	20.63 (4,116.52)	20.38 (18.23)	4.35 (2.68)
Metastases to liver	39	11.28 (8.23–15.46)	11.25 (361.71)	11.18 (8.58)	3.48 (1.82)
Melanocytic naevus	30	30.16 (21.01–43.3)	30.09 (828.53)	29.56 (21.85)	4.89 (3.22)
Tumour pain	18	58.14 (36.32–93.06)	58.05 (974.64)	56.09 (37.84)	5.81 (4.14)
Cancer pain	17	26.23 (16.24–42.37)	26.2 (405.52)	25.80 (17.27)	4.69 (3.02)
Neoplasm	17	6.65 (4.13–10.72)	6.65 (81.24)	6.62 (4.45)	2.73 (1.06)
Squamous cell carcinoma of skin	15	11.74 (7.06–19.52)	11.73 (146.19)	11.65 (7.62)	3.54 (1.88)
Skin papilloma	15	19.86 (11.93–33.05)	19.84 (265.11)	19.61 (12.81)	4.29 (2.63)
Hepatic neoplasm	14	25.76 (15.19–43.68)	25.73 (327.60)	25.35 (16.29)	4.66 (2.99)
Tumour compression	10	173.75 (90.51–333.55)	173.61 (1,551.18)	157.02 (90.98)	7.29 (5.61)
SOC: Nervous system disorders					
Hypersomnia^a^	18	3.24 (2.04–5.15)	3.24 (27.81)	3.23 (2.20)	1.69 (0.03)
SOC: Skin and subcutaneous tissue disorders					
Alopecia	436	11.92 (10.83–13.12)	11.55 (4,182.69)	11.47 (10.59)	3.52 (1.85)
Dry skin	164	5.76 (4.93–6.72)	5.69 (633.93)	5.68 (4.99)	2.51 (0.84)
Palmar-plantar erythrodysaesthesia syndrome	147	31.61 (26.83–37.25)	31.26 (4,226.18)	30.69 (26.75)	4.94 (3.27)
Hyperkeratosis	111	116.02 (95.63–140.76)	115.01 (11,720.58)	107.51 (91.46)	6.75 (5.08)
Blister	60	5.49 (4.26–7.08)	5.47 (218.43)	5.45 (4.41)	2.45 (0.78)
Skin exfoliation	60	3.54 (2.74–4.56)	3.52 (108.34)	3.52 (2.84)	1.81 (0.15)
Hair texture abnormal	45	21.25 (15.83–28.53)	21.18 (854.38)	20.92 (16.35)	4.39 (2.72)
Pain of skin	29	5.19 (3.61–7.48)	5.18 (97.65)	5.17 (3.81)	2.37 (0.70)
Skin disorder	28	3.70 (2.55–5.36)	3.69 (54.92)	3.69 (2.70)	1.88 (0.22)
Skin fissures	21	4.68 (3.05–7.19)	4.68 (60.55)	4.67 (3.26)	2.22 (0.56)
Skin hypertrophy	19	35.77 (22.7–56.38)	35.72 (627.55)	34.98 (23.91)	5.13 (3.46)
Hair colour changes	12	5.17 (2.93–9.12)	5.17 (40.21)	5.15 (3.21)	2.37 (0.70)
Hair growth abnormal	12	11.32 (6.42–19.98)	11.31 (112.05)	11.24 (6.99)	3.49 (1.82)
Sensitive skin	12	4.86 (2.75–8.56)	4.85 (36.60)	4.84 (3.01)	2.28 (0.61)
SOC: Surgical and medical procedures					
Hospitalisation	201	5.62 (4.89–6.46)	5.55 (749.12)	5.53 (4.92)	2.47 (0.8)
Surgery	79	6.72 (5.38–8.38)	6.68 (380.32)	6.66 (5.53)	2.73 (1.07)
Hospice care	17	5.67 (3.52–9.14)	5.67 (65.12)	5.65 (3.79)	2.50 (0.83)
Tumour excision	14	90.05 (52.56–154.27)	89.95 (1,167.15)	85.30 (54.37)	6.41 (4.74)
Therapy change	10	5.05 (2.72–9.4)	5.05 (32.39)	5.04 (3.00)	2.33 (0.67)
SOC: Vascular disorders					
Hypertension	148	3.58 (3.04–4.2)	3.55 (270.72)	3.54 (3.09)	1.82 (0.16)

^a^ , AEs, that are not mentioned in the drug label.

**TABLE 4:** The top 50 signal strength of AEs of ripretinib ranked by the ROR at the PTs level.

PTs	N	ROR (95% two-sided CI)	PRR (χ^2^)	EBGM (EBGM05)	IC (IC025)
SOC: Gastrointestinal disorders					
Tongue haemorrhage	3	15.65 (5.02–48.78)	15.64 (40.73)	15.50 (5.99)	3.95 (2.28)
Gingival bleeding	33	15.59 (11.06–21.97)	15.55 (445.15)	15.41 (11.57)	3.95 (2.28)
SOC: General disorders and administration site conditions					
Adverse event	203	14.56 (12.67–16.74)	14.34 (2,500.71)	14.23 (12.66)	3.83 (2.16)
SOC: Injury, poisoning and procedural complications					
Drain site complication	3	99.94 (31.15–320.67)	99.92 (276.83)	94.21 (35.52)	6.56 (4.85)
Extra dose administered	329	38.16 (34.16–42.63)	37.20 (11,338.8)	36.39 (33.17)	5.19 (3.52)
Underdose	270	21.1 (18.69–23.83)	20.68 (4,997.58)	20.43 (18.46)	4.35 (2.69)
SOC: Investigations					
Blood osmolarity decreased	5	76.29 (31.11–187.07)	76.26 (354.79)	72.90 (34.42)	6.19 (4.5)
Scan abnormal	5	52.66 (21.61–128.34)	52.64 (245.40)	51.03 (24.22)	5.67 (3.99)
Blood magnesium abnormal^a^	4	35.11 (13.04–94.54)	35.10 (129.71)	34.38 (15.01)	5.10 (3.42)
Nutritional condition abnormal	5	33.87 (13.97–82.13)	33.86 (156.20)	33.19 (15.82)	5.05 (3.38)
Granulocyte count increased^a^	3	26.19 (8.37–81.95)	26.18 (71.51)	25.78 (9.93)	4.69 (3.01)
Blood urea abnormal^a^	3	17.12 (5.49–53.41)	17.12 (45.06)	16.95 (6.54)	4.08 (2.41)
Blood chloride decreased^a^	4	16.16 (6.04–43.28)	16.16 (56.32)	16.01 (7.02)	4.00 (2.33)
Computerised tomogram abnormal	4	12.63 (4.72–33.79)	12.63 (42.49)	12.54 (5.50)	3.65 (1.98)
SOC: Metabolism and nutrition disorders					
Weight gain poor	5	11.09 (4.60–26.73)	11.09 (45.58)	11.02 (5.28)	3.46 (1.79)
SOC: Neoplasms benign, malignant and unspecified (incl cysts and polyps)					
Tumour compression	10	173.75 (90.51–333.55)	173.61 (1,551.18)	157.02 (90.98)	7.29 (5.61)
Tumour pain	18	58.14 (36.32–93.06)	58.05 (974.64)	56.09 (37.84)	5.81 (4.14)
Gastric neoplasm	5	42.96 (17.68–104.43)	42.95 (199.60)	41.87 (19.91)	5.39 (3.71)
Melanocytic naevus	30	30.16 (21.01–43.30)	30.09 (828.53)	29.56 (21.85)	4.89 (3.22)
Cancer pain	17	26.23 (16.24–42.37)	26.20 (405.52)	25.80 (17.27)	4.69 (3.02)
Hepatic neoplasm	14	25.76 (15.19–43.68)	25.73 (327.60)	25.35 (16.29)	4.66 (2.99)
Abdominal neoplasm	4	24.10 (8.98–64.68)	24.09 (87.23)	23.75 (10.40)	4.57 (2.89)
Neoplasm progression	223	20.98 (18.36–23.97)	20.63 (4,116.52)	20.38 (18.23)	4.35 (2.68)
Skin papilloma	15	19.86 (11.93–33.05)	19.84 (265.11)	19.61 (12.81)	4.29 (2.63)
Acrochordon	5	18.34 (7.60–44.3)	18.34 (81.05)	18.14 (8.68)	4.18 (2.51)
Spinal cord neoplasm	4	18.34 (6.85–49.15)	18.34 (64.84)	18.14 (7.95)	4.18 (2.51)
Neoplasm skin	5	14.92 (6.19–36.00)	14.92 (64.34)	14.79 (7.08)	3.89 (2.22)
Oncologic complication	3	14.03 (4.50–43.72)	14.03 (35.99)	13.92 (5.38)	3.80 (2.13)
Metastases to peritoneum	7	13.84 (6.57–29.12)	13.83 (82.62)	13.72 (7.36)	3.78 (2.11)
Squamous cell carcinoma of skin	15	11.74 (7.06–19.52)	11.73 (146.19)	11.65 (7.62)	3.54 (1.88)
Gastrointestinal stromal tumour	5	11.74 (4.87–28.31)	11.74 (48.78)	11.66 (5.59)	3.54 (1.87)
Metastases to liver	39	11.28 (8.23–15.46)	11.25 (361.71)	11.18 (8.58)	3.48 (1.82)
Tumour haemorrhage	6	11.18 (5.01–24.96)	11.18 (55.23)	11.11 (5.67)	3.47 (1.80)
SOC: Product Issues					
Product coating issue	8	47.50 (23.52–95.97)	47.48 (353.69)	46.16 (25.63)	5.53 (3.85)
Product shape issue	4	28.03 (10.43–75.31)	28.02 (102.45)	27.56 (12.05)	4.78 (3.11)
SOC: Reproductive system and breast disorders					
Nipple disorder	3	49.97 (15.84–157.64)	49.96 (139.66)	48.5 (18.55)	5.60 (3.91)
SOC: Respiratory, thoracic and mediastinal disorders					
Pleural mass^a^	4	204.06 (72.16–577.09)	204.00 (718.22)	181.44 (76.03)	7.50 (5.77)
SOC: Skin and subcutaneous tissue disorders					
Hyperkeratosis	111	116.02 (95.63–140.76)	115.01 (11,720.58)	107.51 (91.46)	6.75 (5.08)
Ephelides	9	79.88 (40.90–156.00)	79.82 (667.87)	76.15 (43.49)	6.25 (4.57)
Skin hypertrophy	19	35.77 (22.7–56.38)	35.72 (627.55)	34.98 (23.91)	5.13 (3.46)
Palmar-plantar erythrodysaesthesia syndrome	147	31.61 (26.83–37.25)	31.26 (4,226.18)	30.69 (26.75)	4.94 (3.27)
Hair texture abnormal	45	21.25 (15.83–28.53)	21.18 (854.38)	20.92 (16.35)	4.39 (2.72)
Solar lentigo	4	12.80 (4.79–34.25)	12.80 (43.17)	12.71 (5.58)	3.67 (2.00)
Alopecia	436	11.92 (10.83–13.12)	11.55 (4,182.69)	11.47 (10.59)	3.52 (1.85)
Hair growth abnormal	12	11.32 (6.42–19.98)	11.31 (112.05)	11.24 (6.99)	3.49 (1.82)
SOC: Surgical and medical procedures					
Hepatic embolisation	3	288.06 (84.41–983.09)	287.99 (729.29)	244.94 (87.70)	7.94 (6.16)
Tumour excision	14	90.05 (52.56–154.27)	89.95 (1,167.15)	85.30 (54.37)	6.41 (4.74)
Abdominal cavity drainage	5	70.98 (28.99–173.82)	70.95 (330.48)	68.04 (32.16)	6.09 (4.40)
Ostomy bag placement	4	28.89 (10.75–77.66)	28.88 (105.80)	28.40 (12.42)	4.83 (3.15)
Colostomy	5	12.13 (5.03–29.24)	12.12 (50.66)	12.04 (5.77)	3.59 (1.92)

^a^ , AEs, that are not mentioned in the drug label.

**FIGURE 4:**
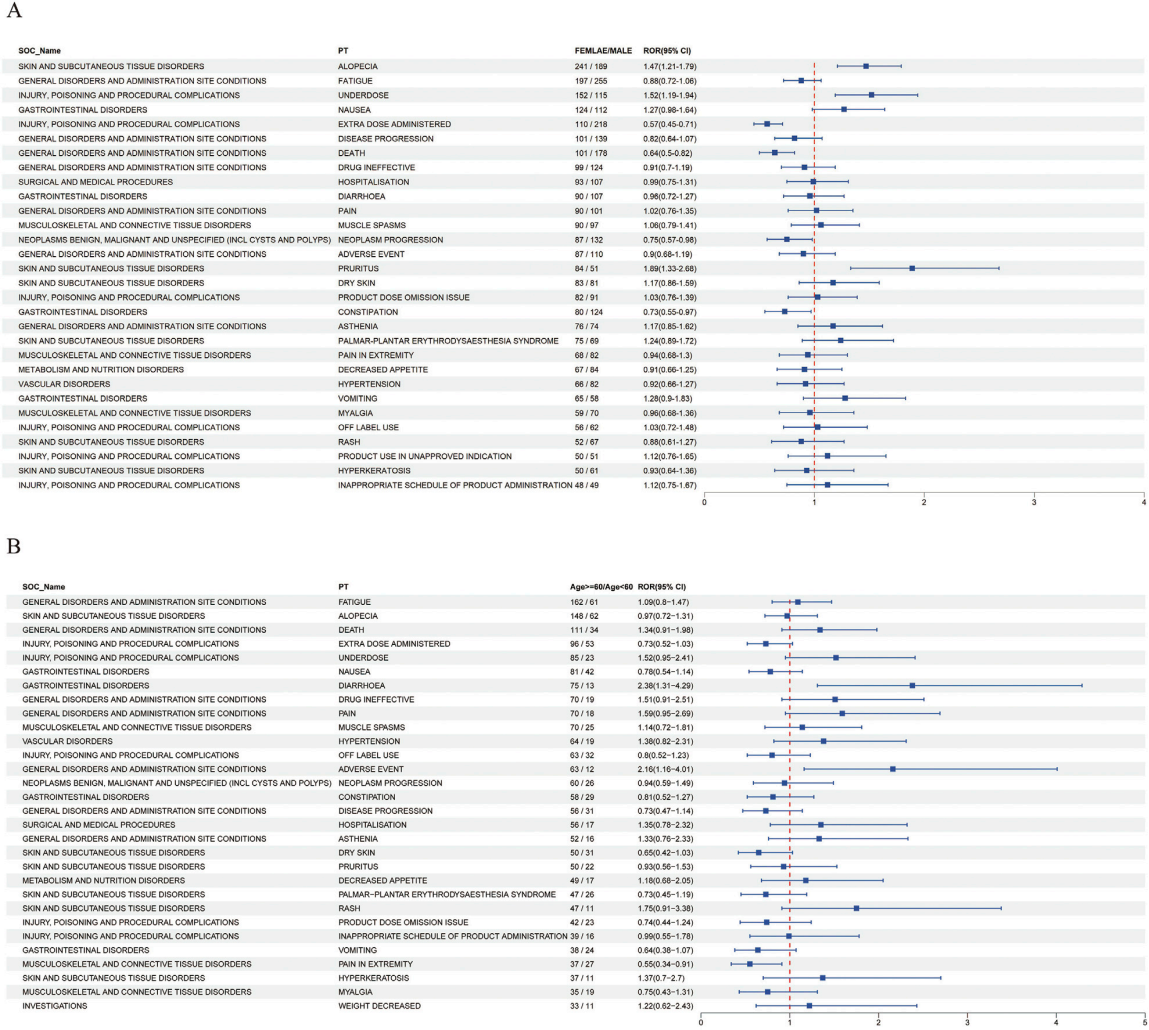
**(A)** Forest plot of gender differences in adverse events at the PT level **(B)** Forest plot of age differences in adverse events at the PT level.

To access whether gender influences AEs associated with ripretinib, the top 30 PTs for female patients were analyzed using the ROR method. Alopecia, underdose, and pruritus were found to be more frequent in females, whereas extra dose administered, death, neoplasm progression, and constipation were more common in males. Figure 4A depicts the results, and Supplementary Table S4 contains all of the data. Prospective studies are planned in the future to further validate the findings of this research.

To evaluate whether age influences AEs associated with ripretinib, the ROR method was used to analyze the top 30 PTs in patients over 60 years old. The results showed statistically significant differences in the occurrence of diarrhoea and pain in extremity between different age groups. Diarrhoea occurred more frequently in patients aged over 60, while pain in extremity was more common in those under 60. These results are shown in Figure 4B, and all data can be found in Supplementary Table S5.

### 3.4 TTO analysis

**FIGURE 5:**
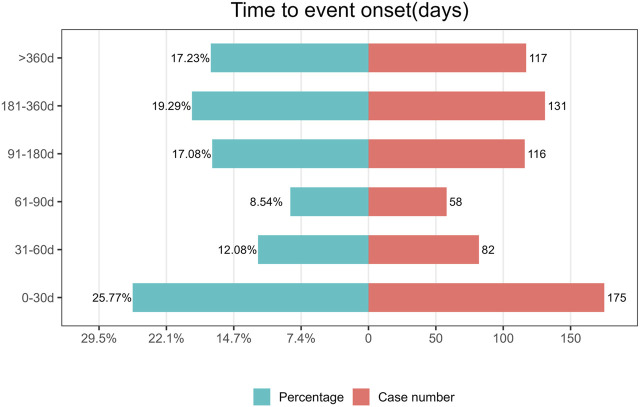
Time to onset of adverse events (AEs) associated with ripretinib.

The TTO of ripretinib-related AEs was determined based on 678 ICSRs with precise onset timings. The median TTO of ripretinib-related AEs was 102 days (29–254 days). Our findings showed that 25.77% of patients experienced an AE within the first month of using ripretinib (n = 175). Notably, patients remained at risk of AEs even after 1 year of ripretinib treatment (n = 117). Figure 5 shows these results.

## 4 Discussion

In recent years, various studies have examined pharmacovigilance analysis for cardiotoxicity, interstitial lung disease, and neuropsychiatric disorders associated with TKIs (Anand et al., 2019; Barbieri et al., 2023; Zhang et al., 2023). Early research on ripretinib primarily focused on clinical trials, clinical efficacy, and literature reviews, with limited attention given to real-world safety evaluations. In this study, we analyzed AEs associated with ripretinib using data from FAERS. Our findings indicate that the most common AEs observed in patients receiving ripretinib were consistent with those reported in clinical trials and drug labels, including alopecia, hypertension, and palmar-plantar erythrodysaesthesia syndrome. In addition, we also identified some unexpected AEs, such as pleural mass, blood magnesium abnormalities, and hepatic lesions. Given the increasing clinical use of ripretinib, our findings provide valuable insights into its safety, particularly in real-world settings.

Using disproportionality analysis, significant SOCs identified included surgical and medical procedures, as well as subcutaneous tissue disorders. The most frequently reported SOCs were general disorders and administration site conditions. Common AEs included alopecia, constipation, muscle spasms, dry skin, decreased appetite, hypertension, palmar-plantar erythrodysaesthesia syndrome, myalgia, and hyperkeratosis. These AEs are consistent with the drug labels and clinical trials (Dhillon, 2020; Zalcberg, 2021; Janku et al., 2022). The most common AE for ripretinib was alopecia (Thirasastr and Somaiah, 2023). In the INVICTUS trial, individuals taking ripretinib experienced alopecia at a rate of exceeding 20% (Fung and Shirley, 2022). One study found that quality of life was equally maintained up to day 1 of treatment cycle 10 in patients treated with ripretinib, regardless of whether or not alopecia occurred (Schöffski et al., 2022).

The product labeling for ripretinib warns of the risk of hypertension associated with the drug and suggests that individuals with uncontrolled hypertension should not initiate treatment. Our data also indicate that ripretinib is more frequently associated with hypertension. Furthermore, hypertension is a rather prevalent AE associated with other TKI medications (Højer Wang et al., 2023). It has been demonstrated to be the outcome of reduced vasodilatory nitric oxide synthesis, decreased prostacyclin production, and increased vasoconstrictor endothelin-1 production. TKI-induced hypertension is dosage-dependent (Ptinopoulou and Sprangers, 2021). Therefore, according to the drug label, blood pressure should be monitored during ripretinib treatment. If symptoms of hypertension develop, ripretinib administration should be suspended and antihypertensive therapy initiated. Once blood pressure is effectively controlled, ripretinib may be resumed with dose adjustments.

Our study also identified several AEs with high signal intensity, including hyperkeratosis, ephelides, palmar-plantar erythrodysaesthesia syndrome, melanocytic naevus, gingival bleeding, and tongue haemorrhage. In the INVICTUS trial, the incidence of palmoplantar erythema syndrome caused by ripretinib was exceeded 20% (Blay et al., 2020). A study that recommended dermatologic evaluation of suspicious skin lesions during ripretinib treatment found that patients who developed squamous cell carcinoma lesions were elderly (mean age 72 years), and the lesions were located in sun-exposed areas (Patel et al., 2021). Squamous cell carcinoma lesions did not exhibit aggressive histopathologic features and were similar to the lowest risk UV-induced lesions (Patel et al., 2021). The pathophysiology of ripretinib induced skin related AEs is not fully understood and requires additional investigation. Ripretinib has been shown to block the vascular endothelial growth factor (VEGF) signaling pathway, potentially slowing wound healing (Dhillon, 2020). Gingival bleeding and tongue haemorrhage were observed in our study, which might be associated with this process (Fung and Shirley, 2022). Furthermore, in a meta-analysis, bleeding events were the most common AEs, and the increased risk of bleeding may have been caused by the co-administration of VEGF inhibitors and antiplatelet medications (Je et al., 2009). These findings highlight the importance for healthcare professionals to monitor for ripretinib-induced bleeding events.

In addition, we detected some unexpected AEs, such as pleural mass, blood magnesium abnormal, prostatomegaly, blood potassium abnormal, hepatic lesion, liver abscess and neovascular age-related macular degeneration. When using ripretinib, healthcare professionals should be aware of the risk of these unexpected AEs occurring in patients to ensure patient safety. So far, there have been no documented reports of these unexpected AEs. Therefore, it is essential to explore the pathogenesis and extent of harm caused by these unexpected AEs. In our study, ripretinib-induced hepatotoxicity was found to include hepatic lesions and liver abscesses. In addition, similar AEs have also been observed with other TKIs (Teo et al., 2013). The onset of these disease typically occurs 1–3 weeks, or even months, after drug administration (Lammert et al., 2008). It has been suggested that TKIs generate toxic metabolites *in vivo*, cause mitochondrial dysfunction, and inhibit glycolysis, which have been described as possible mechanisms (Teo et al., 2013; Paech et al., 2017). For reports of abnormalities in blood potassium and magnesium, it is recommended that electrolyte concentrations be monitored regularly during drug use, and that relevant electrolytes be supplemented promptly when abnormalities are detected. For age-related macular degeneration and dry age-related macular degeneration, patients should be advised to monitor their daily ocular symptoms and seek prompt medical attention if they experience discomfort.

Subgroup analyses by gender revealed that females were at a high risk for alopecia, underdose, and pruritus, while males were at a higher risk for extra dose administration, death, neoplasm progression, and constipation. It has been shown that gender differences affect drug toxicity in colorectal cancer chemotherapy, with a significantly higher risk of alopecia, vomiting, weakness, nausea, and pain events occurring in females compared to males (De Francia et al., 2022). The increased risk of AEs may be attributable to the physiologic characteristics between genders, which warrants further investigation into the underlying mechanisms. Patients aged 60 and above are at an elevated risk of developing diarrhoea, which may be attributed to both the age-related decline in gastrointestinal function and the potential toxicity of pharmacological agents. Severe diarrhoea can result in electrolyte disturbances, which, in turn, can lead to more severe complications, particularly in the elderly population (Reintam Blaser et al., 2015; Hammer, 2021). Consequently, prior to the initiation of treatment, it is essential to inform patients of the potential risk of diarrhoea and to implement vigilant monitoring for its occurrence. Furthermore, it is advisable for patients to modify their dietary habits as a preventive measure and, if necessary, promptly restore electrolyte balance and administer antidiarrheal agents such as loperamide. Patients under the age of 60 may be at a higher risk of experiencing pain in extremities, which could negatively affect their medication adherence. Physicians should manage pain based on its severity, adhering to the principles of stepwise analgesia, in order to optimize the patient’s quality of life and ensure adherence to prescribed therapies. Overall, the findings provide an analysis of AEs related to gender and age, though further validation through future studies is necessary.

We observed that common AEs recorded in previous clinical trials, such as nausea, diarrhea, and abdominal pain, did not appear as positive signals in the disproportionality analysis. This may be due to these AEs being highly prevalent in the FAERS database, which could mask the detection of disproportionality. This is known as the masking effect (Lin et al., 2024). Therefore, an accurate assessment of the safety of ripretinib needs to be combined with more prospective and real-world data.

In this study, we analyzed TTO of AE reports in 678 cases with complete records. The median TTO of AEs in this study was 102 days. Notably, 25.77% of ICSRs occurred within the first month of treatment with ripretinib, while 17.23% occurred after one year of treatment with ripretinib. Therefore, it is important to strengthen the long-term monitoring of AEs during treatment with ripretinib.

Despite our analysis of AEs related to ripretinib in FAERS, this study has some limitations due to the database itself. First, the reports from the FAERS database are spontaneously uploaded by healthcare professionals, pharmacists, consumers, and lawyers, and lack detailed clinical information such as comorbidities, concomitant medications, age, and height, which may affect the accuracy of the results. Second, less severe or common AEs may be underestimated, whereas more severe or rare AEs may be overestimated (Wang et al., 2024). Third, disproportionality analysis only reveals statistical associations and does not establish causality between medications and AEs, which requires confirmation by prospective study. Despite these limitations, our study provides a basis for the safe use of ripretinib.

## 5 Conclusion

In conclusion, this study provides a systematic and comprehensive exploration of ripretinib-related AEs based on the FAERS database. The common AEs detected in this study were consistent with drug labeling and clinical trials, while some unexpected adverse events, such as pleural mass, blood magnesium abnormality, prostatomegaly, and hepatic lesion, were also identified. Additionally, differences in AEs between different genders and age groups were analyzed, and the TTO of adverse events was examined. This study contributes to the safer use of ripretinib by healthcare professionals. Future prospective studies and long-term clinical trials are needed to further validate the findings of this research.

## Data Availability

The original contributions presented in the study are included in the article/Supplementary Material, further inquiries can be directed to the corresponding authors.
