# Supplementary material for: Signal mining and analysis of ripretinib adverse events: a real-world pharmacovigilance analysis based on the FAERS database
Source: Front Pharmacol. 2025 Feb 26;16:1481114. doi: 10.3389/fphar.2025.1481114 (PMC11896992; doi:10.3389/fphar.2025.1481114)
Supplement: Supplementary file 1 [file DataSheet1.docx]

**Signal mining and analysis of ripretinib adverse events: a real-world pharmacovigilance analysis based on the FAERS database**

**Supplementary Table 1. Four major methods used for signal detection**

| **Algorithms** | **Equation** | **Criteria** |
| --- | --- | --- |
| ROR | ROR=ad/b/c | lower limit of 95% CI>1, a≥3 |
|  | 95%CI=e^ln(ROR)±1.96(1/a+1/b+1/c+1/d)^0.5^ |  |
| PRR | PRR=a(c+d)/c/(a+b) | PRR≥2, χ^2^≥4, a≥3 |
|  | χ^2^=[(ad-bc)^2](a+b+c+d)/[(a+b)(c+d)(a+c)(b+d)] |  |
| BCPNN | IC=log_2_a(a+b+c+d)/(a+c)/(a+b) | IC025>0 |
|  | 95%CI= E(IC) ± 2V(IC)^0.5 |  |
| MGPS | EBGM=a(a+b+c+d)/(a+c)/(a+b) | EBGM05>2 |
|  | 95%CI=e^ln(EBGM)±1.64(1/a+1/b+1/c+1/d)^0.5^ |  |

Equation: a, number of reports containing both the target drug and target adverse drug reaction; b, number of reports containing other adverse drug reaction of the target drug; c, number of reports containing the target adverse drug reaction of other drugs; d, number of reports containing other drugs and other adverse drug reactions. 95%CI, 95% confidence interval; χ^2^, chi-squared; IC, information component; IC025, the lower limit of 95% CI of the IC; E(IC), the IC expectations; V(IC), the variance of IC; EBGM, empirical Bayesian geometric mean; EBGM05, the lower limit of 95% CI of EBGM.

**Supplementary Table 2. Signal detection form for gender and age differences**

|  | Target adverse drug event with ripretinib | Other adverse drug events with ripretinib | Sums |
| --- | --- | --- | --- |
| Females/Age>=60 | a | b | a+b |
| Males/Age<60 | c | d | c+d |
| Sums | a+c | b+d | a+b+c+d |

**Supplementary Table 3**

**Ripretinib-related AEs sorted by frequency at PT level**

| SOC | PTs | N | ROR (95% two-sided CI) | PRR (χ^2^) | EBGM (EBGM05) | IC (IC025) |
| --- | --- | --- | --- | --- | --- | --- |
| SKIN AND SUBCUTANEOUS TISSUE DISORDERS | ALOPECIA | 436 | 11.92 ( 10.83 - 13.12 ) | 11.55 ( 4182.69 ) | 11.47 ( 10.59 ) | 3.52 ( 1.85 ) |
| INJURY, POISONING AND PROCEDURAL COMPLICATIONS | EXTRA DOSE ADMINISTERED | 329 | 38.16 ( 34.16 - 42.63 ) | 37.2 ( 11338.8 ) | 36.39 ( 33.17 ) | 5.19 ( 3.52 ) |
| INJURY, POISONING AND PROCEDURAL COMPLICATIONS | UNDERDOSE | 270 | 21.1 ( 18.69 - 23.83 ) | 20.68 ( 4997.58 ) | 20.43 ( 18.46 ) | 4.35 ( 2.69 ) |
| GENERAL DISORDERS AND ADMINISTRATION SITE CONDITIONS | DISEASE PROGRESSION | 246 | 9.69 ( 8.53 - 10.99 ) | 9.52 ( 1867.97 ) | 9.47 ( 8.52 ) | 3.24 ( 1.58 ) |
| NEOPLASMS BENIGN, MALIGNANT AND UNSPECIFIED (INCL CYSTS AND POLYPS) | NEOPLASM PROGRESSION | 223 | 20.98 ( 18.36 - 23.97 ) | 20.63 ( 4116.52 ) | 20.38 ( 18.23 ) | 4.35 ( 2.68 ) |
| GASTROINTESTINAL DISORDERS | CONSTIPATION | 204 | 4.66 ( 4.06 - 5.36 ) | 4.61 ( 576.16 ) | 4.6 ( 4.09 ) | 2.2 ( 0.53 ) |
| GENERAL DISORDERS AND ADMINISTRATION SITE CONDITIONS | ADVERSE EVENT | 203 | 14.56 ( 12.67 - 16.74 ) | 14.34 ( 2500.71 ) | 14.23 ( 12.66 ) | 3.83 ( 2.16 ) |
| SURGICAL AND MEDICAL PROCEDURES | HOSPITALISATION | 201 | 5.62 ( 4.89 - 6.46 ) | 5.55 ( 749.12 ) | 5.53 ( 4.92 ) | 2.47 ( 0.8 ) |
| MUSCULOSKELETAL AND CONNECTIVE TISSUE DISORDERS | MUSCLE SPASMS | 188 | 6.13 ( 5.31 - 7.08 ) | 6.06 ( 792.65 ) | 6.04 ( 5.35 ) | 2.59 ( 0.93 ) |
| SKIN AND SUBCUTANEOUS TISSUE DISORDERS | DRY SKIN | 164 | 5.76 ( 4.93 - 6.72 ) | 5.69 ( 633.93 ) | 5.68 ( 4.99 ) | 2.51 ( 0.84 ) |
| METABOLISM AND NUTRITION DISORDERS | DECREASED APPETITE | 154 | 3.27 ( 2.79 - 3.83 ) | 3.24 ( 238.87 ) | 3.24 ( 2.83 ) | 1.69 ( 0.03 ) |
| VASCULAR DISORDERS | HYPERTENSION | 148 | 3.58 ( 3.04 - 4.2 ) | 3.55 ( 270.72 ) | 3.54 ( 3.09 ) | 1.82 ( 0.16 ) |
| SKIN AND SUBCUTANEOUS TISSUE DISORDERS | PALMAR-PLANTAR ERYTHRODYSAESTHESIA SYNDROME | 147 | 31.61 ( 26.83 - 37.25 ) | 31.26 ( 4226.18 ) | 30.69 ( 26.75 ) | 4.94 ( 3.27 ) |
| MUSCULOSKELETAL AND CONNECTIVE TISSUE DISORDERS | MYALGIA | 133 | 4.88 ( 4.11 - 5.79 ) | 4.83 ( 404.24 ) | 4.82 ( 4.18 ) | 2.27 ( 0.6 ) |
| SKIN AND SUBCUTANEOUS TISSUE DISORDERS | HYPERKERATOSIS | 111 | 116.02 ( 95.63 - 140.76 ) | 115.01 ( 11720.58 ) | 107.51 ( 91.46 ) | 6.75 ( 5.08 ) |
| SURGICAL AND MEDICAL PROCEDURES | SURGERY | 79 | 6.72 ( 5.38 - 8.38 ) | 6.68 ( 380.32 ) | 6.66 ( 5.53 ) | 2.73 ( 1.07 ) |
| SKIN AND SUBCUTANEOUS TISSUE DISORDERS | BLISTER | 60 | 5.49 ( 4.26 - 7.08 ) | 5.47 ( 218.43 ) | 5.45 ( 4.41 ) | 2.45 ( 0.78 ) |
| SKIN AND SUBCUTANEOUS TISSUE DISORDERS | SKIN EXFOLIATION | 60 | 3.54 ( 2.74 - 4.56 ) | 3.52 ( 108.34 ) | 3.52 ( 2.84 ) | 1.81 ( 0.15 ) |
| SKIN AND SUBCUTANEOUS TISSUE DISORDERS | HAIR TEXTURE ABNORMAL | 45 | 21.25 ( 15.83 - 28.53 ) | 21.18 ( 854.38 ) | 20.92 ( 16.35 ) | 4.39 ( 2.72 ) |
| INJURY, POISONING AND PROCEDURAL COMPLICATIONS | PRODUCT DOSE OMISSION IN ERROR | 44 | 4.39 ( 3.26 - 5.9 ) | 4.37 ( 114.3 ) | 4.36 ( 3.41 ) | 2.13 ( 0.46 ) |
| NEOPLASMS BENIGN, MALIGNANT AND UNSPECIFIED (INCL CYSTS AND POLYPS) | METASTASES TO LIVER | 39 | 11.28 ( 8.23 - 15.46 ) | 11.25 ( 361.71 ) | 11.18 ( 8.58 ) | 3.48 ( 1.82 ) |
| GASTROINTESTINAL DISORDERS | GINGIVAL BLEEDING | 33 | 15.59 ( 11.06 - 21.97 ) | 15.55 ( 445.15 ) | 15.41 ( 11.57 ) | 3.95 ( 2.28 ) |
| INVESTIGATIONS | BLOOD IRON DECREASED | 31 | 10.23 ( 7.18 - 14.56 ) | 10.2 ( 255.82 ) | 10.15 ( 7.55 ) | 3.34 ( 1.68 ) |
| INVESTIGATIONS | BLOOD BILIRUBIN INCREASED | 30 | 7.76 ( 5.42 - 11.11 ) | 7.75 ( 175.46 ) | 7.71 ( 5.71 ) | 2.95 ( 1.28 ) |
| NEOPLASMS BENIGN, MALIGNANT AND UNSPECIFIED (INCL CYSTS AND POLYPS) | MELANOCYTIC NAEVUS | 30 | 30.16 ( 21.01 - 43.3 ) | 30.09 ( 828.53 ) | 29.56 ( 21.85 ) | 4.89 ( 3.22 ) |
| SKIN AND SUBCUTANEOUS TISSUE DISORDERS | PAIN OF SKIN | 29 | 5.19 ( 3.61 - 7.48 ) | 5.18 ( 97.65 ) | 5.17 ( 3.81 ) | 2.37 ( 0.7 ) |
| SKIN AND SUBCUTANEOUS TISSUE DISORDERS | SKIN DISORDER | 28 | 3.7 ( 2.55 - 5.36 ) | 3.69 ( 54.92 ) | 3.69 ( 2.7 ) | 1.88 ( 0.22 ) |
| SKIN AND SUBCUTANEOUS TISSUE DISORDERS | SKIN FISSURES | 21 | 4.68 ( 3.05 - 7.19 ) | 4.68 ( 60.55 ) | 4.67 ( 3.26 ) | 2.22 ( 0.56 ) |
| INVESTIGATIONS | RED BLOOD CELL COUNT DECREASED | 21 | 3.27 ( 2.13 - 5.02 ) | 3.26 ( 32.93 ) | 3.26 ( 2.28 ) | 1.7 ( 0.04 ) |
| INVESTIGATIONS | BLOOD POTASSIUM DECREASED* | 19 | 3.51 ( 2.24 - 5.51 ) | 3.51 ( 34.05 ) | 3.51 ( 2.4 ) | 1.81 ( 0.14 ) |
| SKIN AND SUBCUTANEOUS TISSUE DISORDERS | SKIN HYPERTROPHY | 19 | 35.77 ( 22.7 - 56.38 ) | 35.72 ( 627.55 ) | 34.98 ( 23.91 ) | 5.13 ( 3.46 ) |
| NEOPLASMS BENIGN, MALIGNANT AND UNSPECIFIED (INCL CYSTS AND POLYPS) | TUMOUR PAIN | 18 | 58.14 ( 36.32 - 93.06 ) | 58.05 ( 974.64 ) | 56.09 ( 37.84 ) | 5.81 ( 4.14 ) |
| NERVOUS SYSTEM DISORDERS | HYPERSOMNIA* | 18 | 3.24 ( 2.04 - 5.15 ) | 3.24 ( 27.81 ) | 3.23 ( 2.2 ) | 1.69 ( 0.03 ) |
| SURGICAL AND MEDICAL PROCEDURES | HOSPICE CARE | 17 | 5.67 ( 3.52 - 9.14 ) | 5.67 ( 65.12 ) | 5.65 ( 3.79 ) | 2.5 ( 0.83 ) |
| NEOPLASMS BENIGN, MALIGNANT AND UNSPECIFIED (INCL CYSTS AND POLYPS) | CANCER PAIN | 17 | 26.23 ( 16.24 - 42.37 ) | 26.2 ( 405.52 ) | 25.8 ( 17.27 ) | 4.69 ( 3.02 ) |
| NEOPLASMS BENIGN, MALIGNANT AND UNSPECIFIED (INCL CYSTS AND POLYPS) | NEOPLASM | 17 | 6.65 ( 4.13 - 10.72 ) | 6.65 ( 81.24 ) | 6.62 ( 4.45 ) | 2.73 ( 1.06 ) |
| INVESTIGATIONS | BLOOD PRESSURE ABNORMAL | 16 | 3.42 ( 2.09 - 5.59 ) | 3.42 ( 27.31 ) | 3.41 ( 2.26 ) | 1.77 ( 0.1 ) |
| NEOPLASMS BENIGN, MALIGNANT AND UNSPECIFIED (INCL CYSTS AND POLYPS) | SQUAMOUS CELL CARCINOMA OF SKIN | 15 | 11.74 ( 7.06 - 19.52 ) | 11.73 ( 146.19 ) | 11.65 ( 7.62 ) | 3.54 ( 1.88 ) |
| NEOPLASMS BENIGN, MALIGNANT AND UNSPECIFIED (INCL CYSTS AND POLYPS) | SKIN PAPILLOMA | 15 | 19.86 ( 11.93 - 33.05 ) | 19.84 ( 265.11 ) | 19.61 ( 12.81 ) | 4.29 ( 2.63 ) |
| SURGICAL AND MEDICAL PROCEDURES | TUMOUR EXCISION | 14 | 90.05 ( 52.56 - 154.27 ) | 89.95 ( 1167.15 ) | 85.3 ( 54.37 ) | 6.41 ( 4.74 ) |
| NEOPLASMS BENIGN, MALIGNANT AND UNSPECIFIED (INCL CYSTS AND POLYPS) | HEPATIC NEOPLASM | 14 | 25.76 ( 15.19 - 43.68 ) | 25.73 ( 327.6 ) | 25.35 ( 16.29 ) | 4.66 ( 2.99 ) |
| INJURY, POISONING AND PROCEDURAL COMPLICATIONS | PRODUCT ADMINISTRATION INTERRUPTED | 13 | 3.34 ( 1.94 - 5.76 ) | 3.34 ( 21.29 ) | 3.34 ( 2.12 ) | 1.74 ( 0.07 ) |
| SKIN AND SUBCUTANEOUS TISSUE DISORDERS | HAIR COLOUR CHANGES | 12 | 5.17 ( 2.93 - 9.12 ) | 5.17 ( 40.21 ) | 5.15 ( 3.21 ) | 2.37 ( 0.7 ) |
| SKIN AND SUBCUTANEOUS TISSUE DISORDERS | HAIR GROWTH ABNORMAL | 12 | 11.32 ( 6.42 - 19.98 ) | 11.31 ( 112.05 ) | 11.24 ( 6.99 ) | 3.49 ( 1.82 ) |
| SKIN AND SUBCUTANEOUS TISSUE DISORDERS | SENSITIVE SKIN | 12 | 4.86 ( 2.75 - 8.56 ) | 4.85 ( 36.6 ) | 4.84 ( 3.01 ) | 2.28 ( 0.61 ) |
| INVESTIGATIONS | BLOOD SODIUM DECREASED | 12 | 3.72 ( 2.11 - 6.57 ) | 3.72 ( 23.84 ) | 3.72 ( 2.31 ) | 1.89 ( 0.23 ) |
| INVESTIGATIONS | BLOOD GLUCOSE ABNORMAL* | 12 | 3.45 ( 1.96 - 6.08 ) | 3.45 ( 20.84 ) | 3.44 ( 2.14 ) | 1.78 ( 0.12 ) |
| INVESTIGATIONS | BLOOD ALKALINE PHOSPHATASE INCREASED | 11 | 3.59 ( 1.99 - 6.49 ) | 3.59 ( 20.5 ) | 3.58 ( 2.18 ) | 1.84 ( 0.17 ) |
| NEOPLASMS BENIGN, MALIGNANT AND UNSPECIFIED (INCL CYSTS AND POLYPS) | TUMOUR COMPRESSION | 10 | 173.75 ( 90.51 - 333.55 ) | 173.61 ( 1551.18 ) | 157.02 ( 90.98 ) | 7.29 ( 5.61 ) |
| SURGICAL AND MEDICAL PROCEDURES | THERAPY CHANGE | 10 | 5.05 ( 2.72 - 9.4 ) | 5.05 ( 32.39 ) | 5.04 ( 3 ) | 2.33 ( 0.67 ) |
| PRODUCT ISSUES | PRODUCT SOLUBILITY ABNORMAL | 10 | 10.07 ( 5.41 - 18.77 ) | 10.07 ( 81.18 ) | 10.01 ( 5.95 ) | 3.32 ( 1.66 ) |
| SURGICAL AND MEDICAL PROCEDURES | TOOTH EXTRACTION | 9 | 4.38 ( 2.27 - 8.42 ) | 4.37 ( 23.36 ) | 4.36 ( 2.52 ) | 2.13 ( 0.46 ) |
| GENERAL DISORDERS AND ADMINISTRATION SITE CONDITIONS | TENDERNESS | 9 | 4.3 ( 2.23 - 8.27 ) | 4.3 ( 22.71 ) | 4.29 ( 2.48 ) | 2.1 ( 0.43 ) |
| SKIN AND SUBCUTANEOUS TISSUE DISORDERS | EPHELIDES | 9 | 79.88 ( 40.9 - 156 ) | 79.82 ( 667.87 ) | 76.15 ( 43.49 ) | 6.25 ( 4.57 ) |
| RESPIRATORY, THORACIC AND MEDIASTINAL DISORDERS | UPPER-AIRWAY COUGH SYNDROME | 9 | 4.78 ( 2.48 - 9.2 ) | 4.78 ( 26.8 ) | 4.77 ( 2.76 ) | 2.25 ( 0.59 ) |
| HEPATOBILIARY DISORDERS | HEPATIC LESION* | 8 | 9.06 ( 4.52 - 18.15 ) | 9.05 ( 57 ) | 9.01 ( 5.04 ) | 3.17 ( 1.5 ) |
| PRODUCT ISSUES | PRODUCT COATING ISSUE | 8 | 47.5 ( 23.52 - 95.97 ) | 47.48 ( 353.69 ) | 46.16 ( 25.63 ) | 5.53 ( 3.85 ) |
| REPRODUCTIVE SYSTEM AND BREAST DISORDERS | PROSTATOMEGALY* | 8 | 10.65 ( 5.31 - 21.34 ) | 10.64 ( 69.43 ) | 10.58 ( 5.91 ) | 3.4 ( 1.73 ) |
| EAR AND LABYRINTH DISORDERS | EAR DISCOMFORT* | 8 | 3.96 ( 1.98 - 7.93 ) | 3.96 ( 17.64 ) | 3.95 ( 2.21 ) | 1.98 ( 0.32 ) |
| INVESTIGATIONS | BLOOD CALCIUM DECREASED | 8 | 3.78 ( 1.89 - 7.56 ) | 3.78 ( 16.3 ) | 3.77 ( 2.11 ) | 1.91 ( 0.25 ) |
| INVESTIGATIONS | BLOOD ALBUMIN DECREASED* | 8 | 7.31 ( 3.65 - 14.63 ) | 7.3 ( 43.32 ) | 7.27 ( 4.07 ) | 2.86 ( 1.2 ) |
| NEOPLASMS BENIGN, MALIGNANT AND UNSPECIFIED (INCL CYSTS AND POLYPS) | METASTASIS | 8 | 5.43 ( 2.71 - 10.87 ) | 5.43 ( 28.79 ) | 5.41 ( 3.03 ) | 2.44 ( 0.77 ) |
| PRODUCT ISSUES | PRODUCT SIZE ISSUE | 7 | 6.54 ( 3.11 - 13.74 ) | 6.54 ( 32.69 ) | 6.51 ( 3.5 ) | 2.7 ( 1.04 ) |
| NEOPLASMS BENIGN, MALIGNANT AND UNSPECIFIED (INCL CYSTS AND POLYPS) | METASTASES TO PERITONEUM | 7 | 13.84 ( 6.57 - 29.12 ) | 13.83 ( 82.62 ) | 13.72 ( 7.36 ) | 3.78 ( 2.11 ) |
| GASTROINTESTINAL DISORDERS | TONGUE DISCOMFORT | 7 | 6.4 ( 3.04 - 13.44 ) | 6.39 ( 31.72 ) | 6.37 ( 3.42 ) | 2.67 ( 1 ) |
| BLOOD AND LYMPHATIC SYSTEM DISORDERS | INCREASED TENDENCY TO BRUISE | 7 | 4.4 ( 2.09 - 9.23 ) | 4.39 ( 18.3 ) | 4.38 ( 2.36 ) | 2.13 ( 0.47 ) |
| NERVOUS SYSTEM DISORDERS | HYPERAESTHESIA | 6 | 4.32 ( 1.94 - 9.63 ) | 4.32 ( 15.26 ) | 4.31 ( 2.2 ) | 2.11 ( 0.44 ) |
| INVESTIGATIONS | BLOOD PHOSPHORUS DECREASED | 6 | 7.13 ( 3.2 - 15.9 ) | 7.13 ( 31.47 ) | 7.1 ( 3.63 ) | 2.83 ( 1.16 ) |
| NEOPLASMS BENIGN, MALIGNANT AND UNSPECIFIED (INCL CYSTS AND POLYPS) | TUMOUR HAEMORRHAGE | 6 | 11.18 ( 5.01 - 24.96 ) | 11.18 ( 55.23 ) | 11.11 ( 5.67 ) | 3.47 ( 1.8 ) |
| SKIN AND SUBCUTANEOUS TISSUE DISORDERS | SKIN ATROPHY | 6 | 5.08 ( 2.28 - 11.33 ) | 5.08 ( 19.59 ) | 5.07 ( 2.59 ) | 2.34 ( 0.67 ) |
| SURGICAL AND MEDICAL PROCEDURES | COLOSTOMY | 5 | 12.13 ( 5.03 - 29.24 ) | 12.12 ( 50.66 ) | 12.04 ( 5.77 ) | 3.59 ( 1.92 ) |
| SURGICAL AND MEDICAL PROCEDURES | ABDOMINAL CAVITY DRAINAGE | 5 | 70.98 ( 28.99 - 173.82 ) | 70.95 ( 330.48 ) | 68.04 ( 32.16 ) | 6.09 ( 4.4 ) |
| METABOLISM AND NUTRITION DISORDERS | WEIGHT GAIN POOR | 5 | 11.09 ( 4.6 - 26.73 ) | 11.09 ( 45.58 ) | 11.02 ( 5.28 ) | 3.46 ( 1.79 ) |
| INVESTIGATIONS | NUTRITIONAL CONDITION ABNORMAL | 5 | 33.87 ( 13.97 - 82.13 ) | 33.86 ( 156.2 ) | 33.19 ( 15.82 ) | 5.05 ( 3.38 ) |
| NEOPLASMS BENIGN, MALIGNANT AND UNSPECIFIED (INCL CYSTS AND POLYPS) | ACROCHORDON | 5 | 18.34 ( 7.6 - 44.3 ) | 18.34 ( 81.05 ) | 18.14 ( 8.68 ) | 4.18 ( 2.51 ) |
| INVESTIGATIONS | BLOOD POTASSIUM ABNORMAL* | 5 | 9.2 ( 3.82 - 22.17 ) | 9.2 ( 36.34 ) | 9.15 ( 4.39 ) | 3.19 ( 1.53 ) |
| NEOPLASMS BENIGN, MALIGNANT AND UNSPECIFIED (INCL CYSTS AND POLYPS) | GASTROINTESTINAL STROMAL TUMOUR | 5 | 11.74 ( 4.87 - 28.31 ) | 11.74 ( 48.78 ) | 11.66 ( 5.59 ) | 3.54 ( 1.87 ) |
| INVESTIGATIONS | SCAN ABNORMAL | 5 | 52.66 ( 21.61 - 128.34 ) | 52.64 ( 245.4 ) | 51.03 ( 24.22 ) | 5.67 ( 3.99 ) |
| NEOPLASMS BENIGN, MALIGNANT AND UNSPECIFIED (INCL CYSTS AND POLYPS) | NEOPLASM SKIN | 5 | 14.92 ( 6.19 - 36 ) | 14.92 ( 64.34 ) | 14.79 ( 7.08 ) | 3.89 ( 2.22 ) |
| NEOPLASMS BENIGN, MALIGNANT AND UNSPECIFIED (INCL CYSTS AND POLYPS) | GASTRIC NEOPLASM | 5 | 42.96 ( 17.68 - 104.43 ) | 42.95 ( 199.6 ) | 41.87 ( 19.91 ) | 5.39 ( 3.71 ) |
| INVESTIGATIONS | BLOOD OSMOLARITY DECREASED | 5 | 76.29 ( 31.11 - 187.07 ) | 76.26 ( 354.79 ) | 72.9 ( 34.42 ) | 6.19 ( 4.5 ) |
| INFECTIONS AND INFESTATIONS | LIVER ABSCESS* | 5 | 8.07 ( 3.35 - 19.44 ) | 8.07 ( 30.82 ) | 8.04 ( 3.85 ) | 3.01 ( 1.34 ) |
| INVESTIGATIONS | VITAMIN B12 DECREASED | 5 | 7.68 ( 3.19 - 18.49 ) | 7.68 ( 28.9 ) | 7.64 ( 3.66 ) | 2.93 ( 1.27 ) |
| INJURY, POISONING AND PROCEDURAL COMPLICATIONS | INTENTIONAL UNDERDOSE | 5 | 4.85 ( 2.01 - 11.66 ) | 4.85 ( 15.22 ) | 4.83 ( 2.32 ) | 2.27 ( 0.61 ) |
| INVESTIGATIONS | BLOOD CREATININE DECREASED* | 4 | 5.81 ( 2.18 - 15.51 ) | 5.81 ( 15.86 ) | 5.79 ( 2.55 ) | 2.53 ( 0.87 ) |
| NEOPLASMS BENIGN, MALIGNANT AND UNSPECIFIED (INCL CYSTS AND POLYPS) | ABDOMINAL NEOPLASM | 4 | 24.1 ( 8.98 - 64.68 ) | 24.09 ( 87.23 ) | 23.75 ( 10.4 ) | 4.57 ( 2.89 ) |
| PRODUCT ISSUES | PRODUCT SHAPE ISSUE | 4 | 28.03 ( 10.43 - 75.31 ) | 28.02 ( 102.45 ) | 27.56 ( 12.05 ) | 4.78 ( 3.11 ) |
| NEOPLASMS BENIGN, MALIGNANT AND UNSPECIFIED (INCL CYSTS AND POLYPS) | SPINAL CORD NEOPLASM | 4 | 18.34 ( 6.85 - 49.15 ) | 18.34 ( 64.84 ) | 18.14 ( 7.95 ) | 4.18 ( 2.51 ) |
| GASTROINTESTINAL DISORDERS | GASTROINTESTINAL PERFORATION* | 4 | 5.77 ( 2.16 - 15.41 ) | 5.77 ( 15.73 ) | 5.75 ( 2.53 ) | 2.52 ( 0.86 ) |
| INVESTIGATIONS | COMPUTERISED TOMOGRAM ABNORMAL | 4 | 12.63 ( 4.72 - 33.79 ) | 12.63 ( 42.49 ) | 12.54 ( 5.5 ) | 3.65 ( 1.98 ) |
| INVESTIGATIONS | BLOOD MAGNESIUM ABNORMAL* | 4 | 35.11 ( 13.04 - 94.54 ) | 35.1 ( 129.71 ) | 34.38 ( 15.01 ) | 5.1 ( 3.42 ) |
| SURGICAL AND MEDICAL PROCEDURES | OSTOMY BAG PLACEMENT | 4 | 28.89 ( 10.75 - 77.66 ) | 28.88 ( 105.8 ) | 28.4 ( 12.42 ) | 4.83 ( 3.15 ) |
| INVESTIGATIONS | PROTEIN TOTAL DECREASED* | 4 | 7.67 ( 2.87 - 20.49 ) | 7.67 ( 23.1 ) | 7.64 ( 3.36 ) | 2.93 ( 1.26 ) |
| INVESTIGATIONS | BLOOD CHLORIDE DECREASED* | 4 | 16.16 ( 6.04 - 43.28 ) | 16.16 ( 56.32 ) | 16.01 ( 7.02 ) | 4 ( 2.33 ) |
| SKIN AND SUBCUTANEOUS TISSUE DISORDERS | SOLAR LENTIGO | 4 | 12.8 ( 4.79 - 34.25 ) | 12.8 ( 43.17 ) | 12.71 ( 5.58 ) | 3.67 ( 2 ) |
| GASTROINTESTINAL DISORDERS | GLOSSITIS | 4 | 7.03 ( 2.63 - 18.77 ) | 7.03 ( 20.59 ) | 7 ( 3.08 ) | 2.81 ( 1.14 ) |
| PSYCHIATRIC DISORDERS | SOMATIC SYMPTOM DISORDER | 4 | 10.17 ( 3.81 - 27.19 ) | 10.17 ( 32.86 ) | 10.11 ( 4.44 ) | 3.34 ( 1.67 ) |
| RESPIRATORY, THORACIC AND MEDIASTINAL DISORDERS | PLEURAL MASS* | 4 | 204.06 ( 72.16 - 577.09 ) | 204 ( 718.22 ) | 181.44 ( 76.03 ) | 7.5 ( 5.77 ) |
| INVESTIGATIONS | MEAN CELL VOLUME INCREASED | 4 | 5.87 ( 2.2 - 15.68 ) | 5.87 ( 16.11 ) | 5.85 ( 2.57 ) | 2.55 ( 0.88 ) |
| GASTROINTESTINAL DISORDERS | TONGUE HAEMORRHAGE | 3 | 15.65 ( 5.02 - 48.78 ) | 15.64 ( 40.73 ) | 15.5 ( 5.99 ) | 3.95 ( 2.28 ) |
| GASTROINTESTINAL DISORDERS | TONGUE ULCERATION | 3 | 5.5 ( 1.77 - 17.09 ) | 5.5 ( 11.01 ) | 5.49 ( 2.12 ) | 2.46 ( 0.79 ) |
| SURGICAL AND MEDICAL PROCEDURES | HEPATIC EMBOLISATION | 3 | 288.06 ( 84.41 - 983.09 ) | 287.99 ( 729.29 ) | 244.94 ( 87.7 ) | 7.94 ( 6.16 ) |
| REPRODUCTIVE SYSTEM AND BREAST DISORDERS | NIPPLE DISORDER | 3 | 49.97 ( 15.84 - 157.64 ) | 49.96 ( 139.66 ) | 48.5 ( 18.55 ) | 5.6 ( 3.91 ) |
| NEOPLASMS BENIGN, MALIGNANT AND UNSPECIFIED (INCL CYSTS AND POLYPS) | ONCOLOGIC COMPLICATION | 3 | 14.03 ( 4.5 - 43.72 ) | 14.03 ( 35.99 ) | 13.92 ( 5.38 ) | 3.8 ( 2.13 ) |
| GASTROINTESTINAL DISORDERS | INTRA-ABDOMINAL FLUID COLLECTION | 3 | 6.26 ( 2.01 - 19.46 ) | 6.26 ( 13.21 ) | 6.24 ( 2.42 ) | 2.64 ( 0.97 ) |
| INVESTIGATIONS | BLOOD ELECTROLYTES DECREASED | 3 | 9.97 ( 3.21 - 31.03 ) | 9.97 ( 24.07 ) | 9.92 ( 3.84 ) | 3.31 ( 1.64 ) |
| INVESTIGATIONS | SERUM FERRITIN DECREASED | 3 | 7.02 ( 2.26 - 21.81 ) | 7.01 ( 15.4 ) | 6.99 ( 2.71 ) | 2.8 ( 1.14 ) |
| REPRODUCTIVE SYSTEM AND BREAST DISORDERS | TESTICULAR PAIN* | 3 | 5.91 ( 1.9 - 18.38 ) | 5.91 ( 12.2 ) | 5.9 ( 2.28 ) | 2.56 ( 0.89 ) |
| INJURY, POISONING AND PROCEDURAL COMPLICATIONS | DRAIN SITE COMPLICATION | 3 | 99.94 ( 31.15 - 320.67 ) | 99.92 ( 276.83 ) | 94.21 ( 35.52 ) | 6.56 ( 4.85 ) |
| GASTROINTESTINAL DISORDERS | SMALL INTESTINAL PERFORATION* | 3 | 9.02 ( 2.9 - 28.05 ) | 9.02 ( 21.26 ) | 8.97 ( 3.47 ) | 3.17 ( 1.49 ) |
| EYE DISORDERS | DRY AGE-RELATED MACULAR DEGENERATION* | 3 | 5.86 ( 1.89 - 18.22 ) | 5.86 ( 12.06 ) | 5.85 ( 2.26 ) | 2.55 ( 0.88 ) |
| EYE DISORDERS | NEOVASCULAR AGE-RELATED MACULAR DEGENERATION* | 3 | 6.84 ( 2.2 - 21.26 ) | 6.84 ( 14.89 ) | 6.81 ( 2.64 ) | 2.77 ( 1.1 ) |
| INVESTIGATIONS | BLOOD UREA ABNORMAL* | 3 | 17.12 ( 5.49 - 53.41 ) | 17.12 ( 45.06 ) | 16.95 ( 6.54 ) | 4.08 ( 2.41 ) |
| SKIN AND SUBCUTANEOUS TISSUE DISORDERS | PRECANCEROUS SKIN LESION | 3 | 10.74 ( 3.45 - 33.43 ) | 10.74 ( 26.32 ) | 10.67 ( 4.13 ) | 3.42 ( 1.74 ) |
| INVESTIGATIONS | GRANULOCYTE COUNT INCREASED* | 3 | 26.19 ( 8.37 - 81.95 ) | 26.18 ( 71.51 ) | 25.78 ( 9.93 ) | 4.69 ( 3.01 ) |
| INVESTIGATIONS | MEAN CELL HAEMOGLOBIN INCREASED | 3 | 6.08 ( 1.96 - 18.88 ) | 6.07 ( 12.67 ) | 6.06 ( 2.34 ) | 2.6 ( 0.93 ) |

**Supplementary Table 4. Analysis of subgroup differences between males and females**

| PT | a | b | c | d | ROR(95%CI) | P_value |
| --- | --- | --- | --- | --- | --- | --- |
| ALOPECIA | 241 | 5631 | 189 | 6500 | 1.47(1.21-1.79) | 0.00 |
| FATIGUE | 197 | 5675 | 255 | 6434 | 0.88(0.72-1.06) | 0.19 |
| UNDERDOSE | 152 | 5720 | 115 | 6574 | 1.52(1.19-1.94) | 0.00 |
| NAUSEA | 124 | 5748 | 112 | 6577 | 1.27(0.98-1.64) | 0.08 |
| EXTRA DOSE ADMINISTERED | 110 | 5762 | 218 | 6471 | 0.57(0.45-0.71) | 0.00 |
| DISEASE PROGRESSION | 101 | 5771 | 139 | 6550 | 0.82(0.64-1.07) | 0.16 |
| DEATH | 101 | 5771 | 178 | 6511 | 0.64(0.5-0.82) | 0.00 |
| DRUG INEFFECTIVE | 99 | 5773 | 124 | 6565 | 0.91(0.7-1.19) | 0.52 |
| HOSPITALISATION | 93 | 5779 | 107 | 6582 | 0.99(0.75-1.31) | 1.00 |
| DIARRHOEA | 90 | 5782 | 107 | 6582 | 0.96(0.72-1.27) | 0.82 |
| PAIN | 90 | 5782 | 101 | 6588 | 1.02(0.76-1.35) | 0.98 |
| MUSCLE SPASMS | 90 | 5782 | 97 | 6592 | 1.06(0.79-1.41) | 0.76 |
| NEOPLASM PROGRESSION | 87 | 5785 | 132 | 6557 | 0.75(0.57-0.98) | 0.04 |
| ADVERSE EVENT | 87 | 5785 | 110 | 6579 | 0.9(0.68-1.19) | 0.51 |
| PRURITUS | 84 | 5788 | 51 | 6638 | 1.89(1.33-2.68) | 0.00 |
| DRY SKIN | 83 | 5789 | 81 | 6608 | 1.17(0.86-1.59) | 0.36 |
| PRODUCT DOSE OMISSION ISSUE | 82 | 5790 | 91 | 6598 | 1.03(0.76-1.39) | 0.92 |
| CONSTIPATION | 80 | 5792 | 124 | 6565 | 0.73(0.55-0.97) | 0.04 |
| ASTHENIA | 76 | 5796 | 74 | 6615 | 1.17(0.85-1.62) | 0.38 |
| PALMAR-PLANTAR ERYTHRODYSAESTHESIA SYNDROME | 75 | 5797 | 69 | 6620 | 1.24(0.89-1.72) | 0.23 |
| PAIN IN EXTREMITY | 68 | 5804 | 82 | 6607 | 0.94(0.68-1.3) | 0.79 |
| DECREASED APPETITE | 67 | 5805 | 84 | 6605 | 0.91(0.66-1.25) | 0.61 |
| HYPERTENSION | 66 | 5806 | 82 | 6607 | 0.92(0.66-1.27) | 0.66 |
| VOMITING | 65 | 5807 | 58 | 6631 | 1.28(0.9-1.83) | 0.20 |
| MYALGIA | 59 | 5813 | 70 | 6619 | 0.96(0.68-1.36) | 0.89 |
| OFF LABEL USE | 56 | 5816 | 62 | 6627 | 1.03(0.72-1.48) | 0.95 |
| RASH | 52 | 5820 | 67 | 6622 | 0.88(0.61-1.27) | 0.56 |
| PRODUCT USE IN UNAPPROVED INDICATION | 50 | 5822 | 51 | 6638 | 1.12(0.76-1.65) | 0.65 |
| HYPERKERATOSIS | 50 | 5822 | 61 | 6628 | 0.93(0.64-1.36) | 0.79 |
| INAPPROPRIATE SCHEDULE OF PRODUCT ADMINISTRATION | 48 | 5824 | 49 | 6640 | 1.12(0.75-1.67) | 0.66 |

**Supplementary Table 5.**

**Analysis of subgroup differences between patients over and under 60 years old**

| PT | a | b | c | d | ROR(95%CI) | P_value |
| --- | --- | --- | --- | --- | --- | --- |
| FATIGUE | 162 | 3939 | 61 | 1610 | 1.09(0.8-1.47) | 0.65 |
| ALOPECIA | 148 | 3953 | 62 | 1609 | 0.97(0.72-1.31) | 0.91 |
| DEATH | 111 | 3990 | 34 | 1637 | 1.34(0.91-1.98) | 0.17 |
| EXTRA DOSE ADMINISTERED | 96 | 4005 | 53 | 1618 | 0.73(0.52-1.03) | 0.09 |
| UNDERDOSE | 85 | 4016 | 23 | 1648 | 1.52(0.95-2.41) | 0.10 |
| NAUSEA | 81 | 4020 | 42 | 1629 | 0.78(0.54-1.14) | 0.24 |
| DIARRHOEA | 75 | 4026 | 13 | 1658 | 2.38(1.31-4.29) | 0.01 |
| DRUG INEFFECTIVE | 70 | 4031 | 19 | 1652 | 1.51(0.91-2.51) | 0.14 |
| PAIN | 70 | 4031 | 18 | 1653 | 1.59(0.95-2.69) | 0.10 |
| MUSCLE SPASMS | 70 | 4031 | 25 | 1646 | 1.14(0.72-1.81) | 0.65 |
| HYPERTENSION | 64 | 4037 | 19 | 1652 | 1.38(0.82-2.31) | 0.27 |
| OFF LABEL USE | 63 | 4038 | 32 | 1639 | 0.8(0.52-1.23) | 0.36 |
| ADVERSE EVENT | 63 | 4038 | 12 | 1659 | 2.16(1.16-4.01) | 0.02 |
| NEOPLASM PROGRESSION | 60 | 4041 | 26 | 1645 | 0.94(0.59-1.49) | 0.89 |
| CONSTIPATION | 58 | 4043 | 29 | 1642 | 0.81(0.52-1.27) | 0.43 |
| DISEASE PROGRESSION | 56 | 4045 | 31 | 1640 | 0.73(0.47-1.14) | 0.21 |
| HOSPITALISATION | 56 | 4045 | 17 | 1654 | 1.35(0.78-2.32) | 0.35 |
| ASTHENIA | 52 | 4049 | 16 | 1655 | 1.33(0.76-2.33) | 0.39 |
| DRY SKIN | 50 | 4051 | 31 | 1640 | 0.65(0.42-1.03) | 0.08 |
| PRURITUS | 50 | 4051 | 22 | 1649 | 0.93(0.56-1.53) | 0.86 |
| DECREASED APPETITE | 49 | 4052 | 17 | 1654 | 1.18(0.68-2.05) | 0.66 |
| PALMAR-PLANTAR ERYTHRODYSAESTHESIA SYNDROME | 47 | 4054 | 26 | 1645 | 0.73(0.45-1.19) | 0.26 |
| RASH | 47 | 4054 | 11 | 1660 | 1.75(0.91-3.38) | 0.12 |
| PRODUCT DOSE OMISSION ISSUE | 42 | 4059 | 23 | 1648 | 0.74(0.44-1.24) | 0.31 |
| INAPPROPRIATE SCHEDULE OF PRODUCT ADMINISTRATION | 39 | 4062 | 16 | 1655 | 0.99(0.55-1.78) | 1.00 |
| VOMITING | 38 | 4063 | 24 | 1647 | 0.64(0.38-1.07) | 0.12 |
| PAIN IN EXTREMITY | 37 | 4064 | 27 | 1644 | 0.55(0.34-0.91) | 0.03 |
| HYPERKERATOSIS | 37 | 4064 | 11 | 1660 | 1.37(0.7-2.7) | 0.44 |
| MYALGIA | 35 | 4066 | 19 | 1652 | 0.75(0.43-1.31) | 0.39 |
| WEIGHT DECREASED | 33 | 4068 | 11 | 1660 | 1.22(0.62-2.43) | 0.68 |
